# Post-surgical complications of median nerve release at the wrist level

**DOI:** 10.11604/pamj.2020.36.173.23685

**Published:** 2020-07-10

**Authors:** Julia Brasileiro de Faria Cavalcante, Pedro Nogarotto Cembraneli, Renata Brasileiro de Faria Cavalcante, Volmer Fernandes Valente Junior, José Edison da Silva Cavalcante

**Affiliations:** 1 Medical Sciences Course, Health Sciences School, Faculdade Ceres (FACERES), São José do Rio Preto, SP, Brazil,; 2 Neurosurgeon, Member of the Brazilian Society of Neurosurgery, Santa Mônica Hospital, Goiânia, GO, Brazil,; 3 Neurosurgeon, PhD in Neurosurgery, Member of the Brazilian Society of Neurosurgery, Professor at Santa Mônica Hospital, Goiânia, GO, Brazil

**Keywords:** Carpal tunnel syndrome, antisepsis, surgical wound infection

## Abstract

Carpal tunnel syndrome is a set of signs and symptoms caused by compression of the median nerve as it travels through the wrist at the carpal tunnel. The diagnosis is clinical and based on the presence of characteristic signs and symptoms. Proper nonsurgical treatment can stop the progression of this disorder and prevent the development of permanent disability. Surgical treatment may be indicated to patients with complications rated as moderate to severe. Although the surgery is relatively simple, basic antisepsis care before, during, and after the procedure, and guidance of patients for the management of wound hygiene upon discharge, make recovery more secure and prevent disabling sequelae. We report a case of a patient that had infection, edema, and temporary loss of flexibility of the fingers after a surgical procedure to release the median nerve.

## Introduction

Carpal tunnel syndrome (CTS) is a set of signs and symptoms caused by compression of the median nerve as it travels through the wrist at the carpal tunnel [1]. It is the most frequent focal compressive mononeuropathy observed in clinical practice and its pathophysiology has multifactorial origin. Patients present with pain or paresthesia (numbness and tingling) in a distribution that includes the median nerve territory, with involvement of the first three digits and the radial half of the fourth digit. The prevalence of CTS ranges from 1% to 5% in the general population, in a 3: 1 female to male ratio. The major risk factors involved are: obesity, female gender, other comorbidities, and genetic predisposition [2, 3].

## Patient and observation

**Figure 1:**
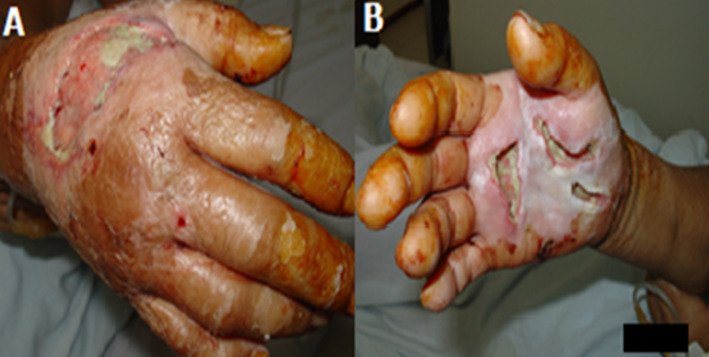
surgical site infection, with inflammation, edema, and local necrosis in the right hand after surgery to release the median nerve. (A) dorsal view; (B) palmar view

A 40-year-old male patient reported pain and paresthesia in his right hand in the last 2 months, associated with weakness in the apprehension of objects and difficulty to work. The neurological examination showed a decrease in the sensitivity of the thumb, index, middle finger, and radial half of the ring finger, as well as positive Tinel´s sign. Electromyography revealed motor and sensory impairment in the median nerve territory. The patient was initially treated with anti-inflammatories and physical therapy, but he did not have improvement. He underwent surgery to release the median nerve by transecting the flexor retinaculum. A few days after hospital discharge, the patient had infection, soft-tissue swelling/edema, heat, pain, and temporary loss of flexibility of the fingers (Figure 1A and B). He was sent back to hospital for surgical wound cleaning and care. The patient was also treated with antibiotics and 2 months of physical therapy. After that, he had some improvement, with partial recovery of the motility of the fingers and wrist, but it was impossible to return to work.

## Discussion

The carpal tunnel is formed by the transverse carpal ligament (flexor retinaculum) superiorly and the carpal bones inferiorly. The median nerve, accompanied by the nine flexor tendons of the forearm musculature, must pass through this anatomical tunnel. The compression of the median nerve can cause ischemia and mechanical nerve interruption [3]. The median nerve emerges from the brachial plexus in the arm with contributions from C6, C7, C8, and T1 nerve roots. C6-C7 nerve roots provide the medium sensory fibers that supply the sensation to the thenar branch and to the first three and a half digits of the hand. C8-T1 nerve roots supply the motor fibers to the muscles of the forearm and hand that are innervated by the median nerve [4]. Although the sensory symptoms of CTS are generally limited to the innervated middle fingers, significant variability has been observed. Pain and paresthesia may be located on the wrist or involve the entire hand. It is not uncommon that sensory symptoms radiate near the forearm, but they less frequently radiate above the elbow to the shoulder, and do not affect the neck [5]. CTS symptoms are often caused by activities that involve flexing or extending the wrist or raising the arms such as driving, reading, typing, and holding the phone [6].

CTS diagnosis is clinical and based on the presence of characteristic signs and symptoms. Provocative maneuvers are used to assist in diagnosis such as: Phalen´s test, Tinel´s sign, wrist flexion with fingers extended, wrist flexion with fingers flexed, wrist extension, combined wrist extension/median nerve pressure, and combined wrist flexion/median nerve pressure [7]. The electrodiagnostic test can be useful in cases the clinical diagnosis is uncertain. It is also useful to assess the severity of nerve compression and assist in decisions related to surgical interventions [8]. Proper nonsurgical treatment can stop the progression of this disorder and prevent the development of a permanent disability. Conservative therapy may be sufficient, although many patients need surgery. Surgical treatment may involve an open or endoscopic technique. The objective of any approach is to decrease the pressure on the median nerve in the wrist, dividing the transverse carpal ligament and the antebrachial fascia [9]. The endoscopic technique brings some advantages such as less surgical trauma, less inflammatory reaction, resulting in an earlier return to usual activities, an average of 8 days sooner than the patients that undergo traditional decompression [10]. The main complications of surgical carpal tunnel release described in the literature are: incomplete release of the transverse carpal ligament (the most frequent), lesion of the median nerve, especially the palmar cutaneous branch, infection, anterior subluxation of the median nerve, and nerve adhesion in the healing region [1,9]. In general, the results of surgical decompression of the median nerve are excellent, with a success rate greater than 90% and a postoperative morbidity of less than 3%. Apparently, the success rates, complications and therapeutic failure rates are similar for traditional and endoscopic surgical treatment [1,9,11].

## Conclusion

Although the surgical approach of CTS is relatively simple, basic antisepsis care before, during, and after the procedure, and guidance of patients for the management of wound hygiene upon discharge, make recovery safer. Therefore, patients should be empowered to self-management by monitoring and treating their own wounds. This could avoid disabling sequelae, as in the case presented, and prevent early retirement of these patients.
